# Supplementary material for: Primary Feline Tauopathy: Clinical, Morphological, Immunohistochemical, and Genetic Studies
Source: Animals (Basel). 2023 Sep 21;13(18):2985. doi: 10.3390/ani13182985 (PMC10525166; doi:10.3390/ani13182985)
Supplement: Supplementary file 1 [file animals-13-02985-s001.zip › animals-2542836-supplementary.pdf]

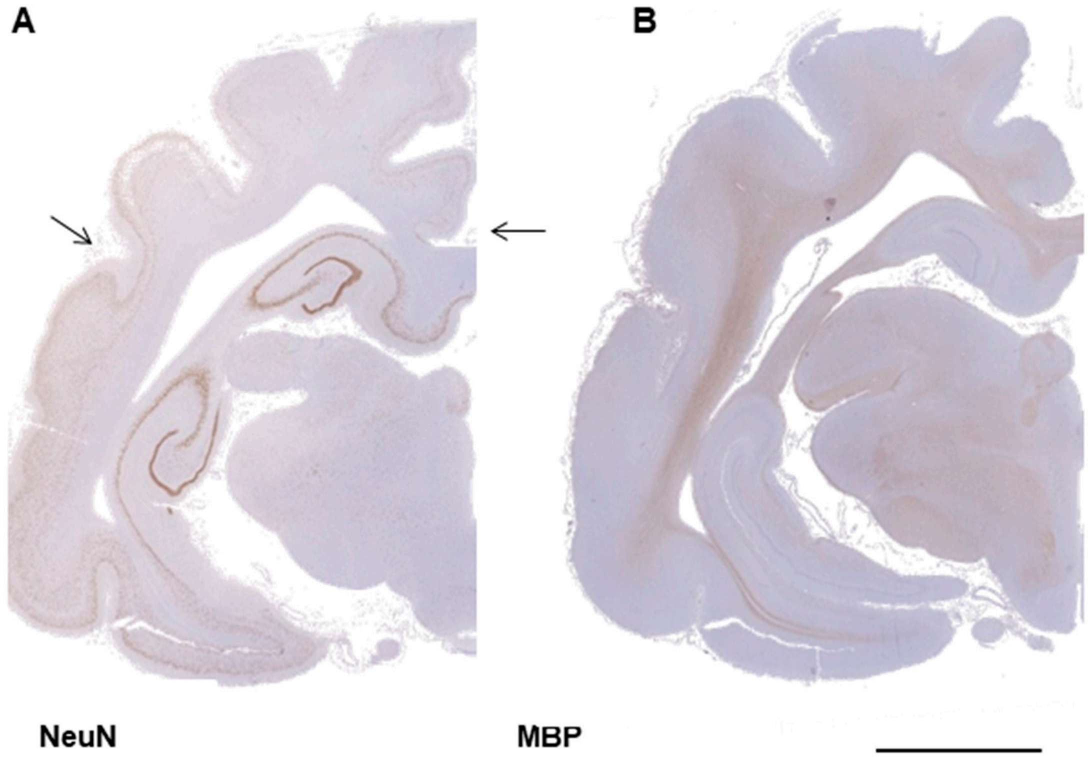

Figure S1. Coronal sections of the telencephalon at the level of the thalamus showing neuron loss, as revealed with the neuronal marker NeuN (A) in the cortical region between thin arrows, and myelin pallor in the subcortical white matter of the parietal lobe, as seen with myelin basic protein (MBP) immunohistochemistry (B). Paraffin sections, bar = 5 mm.

Table S1. Read mapping stats for each sample after targeted capturing of the MAPT locus. The sample 19_S15, in bold, corresponds to the study case.

Sample	Counts	Bases covered	Size locus	% of coverage	Mean coverage
10_S8	23567	96937	98383	0.9853	12.4421
11_S9	25278	96875	98383	0.9847	14.0865
13_S10	19673	96640	98383	0.9823	10.2134
15_S11	15650	95391	98383	0.9696	6.9959
16_S12	20405	95931	98383	0.9751	8.5969
17_S13	39385	97233	98383	0.9883	19.9416
18_S14	36036	97133	98383	0.9873	21.8418
**19_S15**	**28011**	**97026**	**98383**	**0.9862**	**17.0481**
1_S1	29050	97000	98383	0.9859	16.0283
2_S2	23800	96701	98383	0.9829	13.8005
3_S3	30375	97031	98383	0.9863	15.6568
4_S4	12773	93730	98383	0.9527	5.2694
5_S16	18669	95831	98383	0.9741	7.6906
6_S5	3735	63526	98383	0.6457	1.3193
7_S6	9622	93084	98383	0.9461	4.2409
9_S7	28202	97226	98383	0.9882	16.8294

Table S2. Results of the variant calling performed by GATK and VarDict.

	**All samples**		**Case's private variants**	
	**VArDict**	**GATK**	**VArDict**	**GATK**
**5'UTR**	2	2	0	0
**exonic**	33	15	0	0
**intronic**	2310	1531	37*	3
**splicing**	1	1	0	0
**downstream**	14	11	0	0
TOTAL	**2360**	**1560**	**37**	**3**

*** One position contains a different variant in ENSEMBL**
